# Transmission Strategy Modulates Parasite Biogeography in an Island‐Colonising Bird

**DOI:** 10.1111/mec.70659

**Published:** 2026-11-13

**Authors:** Sarah Nichols, Andrea Estandía, Fiona Robertson, Bruce C. Robertson, Beth Okamura, Sonya M. Clegg

**Affiliations:** ^1^ Department of Biology University of Oxford Oxford UK; ^2^ Life Sciences Natural History Museum London UK; ^3^ Department of Zoology Otago University Dunedin New Zealand; ^4^ School of Environment and Science, Griffith University Nathan Queensland Australia

**Keywords:** avian parasite, genomic tools, insect vectors, island biogeography, parasite dispersal, transmission strategy

## Abstract

Parasites occur in every ecosystem, although their dispersal is often constrained by the availability of hosts or vectors. Here, we explore how variation in parasite life history traits, particularly transmission strategy and life cycle complexity, may influence their distributions. Specifically, we test whether a variety of parasites adhere to the rules of island biogeography and whether their distributions vary with life history traits. We utilised broad‐spectrum parasite detection from existing Whole Genome Sequence (WGS) data to characterise parasites with varying transmission strategies from the blood of a passerine bird, the silvereye ( *Zosterops lateralis* ), sampled across 25 islands in the South Pacific and from five of the states in mainland Australia. Overall, parasite richness was higher on mainland Australia compared to islands and decreased with distance of islands from the Australian continent. However, these patterns were dependent on transmission strategy. For parasites transmitted by flying insect vectors, richness decreased on islands compared to the mainland. However, increasing isolation from the mainland among islands had little further impact. On the other hand, richness declined sharply with increasing distance from the mainland for environmentally transmitted parasites and complex life cycle parasites that use intermediate hosts other than ticks or flying insect vectors. While islands may act as an initial barrier to colonisation for parasites relying on flying insect vectors, their highly dispersive intermediate hosts may subsequently reduce the impact of isolation distance on richness. Our work underscores the importance of considering parasite life histories and their transmission strategies for understanding the processes that shape parasite communities on islands.

## 1 Introduction

Parasitic taxa can account for up to 40% of animal biodiversity in an ecosystem (Lafferty, Dobson, and Kuris 2006; Lafferty, Hechinger, et al. 2006; Kuris 2008) and can thus have profound and varied impacts on ecosystem dynamics (Dobson et al. 2008). They may influence inter‐ and intra‐specific interactions, diversification rates and ultimately global patterns of biodiversity (Ricklefs 2010; Tompkins et al. 2011; Morand 2015; Friesen et al. 2020). Therefore, characterising the biogeographic patterns of parasites is important. However, parasites rely on hosts for essential resources (Kuris et al. 1980; Poulin et al. 2011) and their distributions are subsequently dictated by host availability and movement. Parasite dispersal can be further complicated by their own life histories, including transmission strategy. How such life history features influence the biogeographic distributions of parasites is unclear and exacerbated by our generally poor knowledge of parasite diversity (Okamura et al. 2018; Carlson et al. 2020) and difficulty in defining the geographic boundaries of their distributions (Pappalardo et al. 2020). To address these limitations, application of molecular methods to provide broad‐spectrum characterisation of parasite communities in an island biogeography framework offers a powerful means for advancing our understanding of how life history traits shape parasite distributions.

The rules of island biogeography are based on the premise that a mainland (e.g., a continental region or large land mass) acts as a source for islands. A subset of species is expected to colonise and persist on islands, resulting in lower equilibrium species richness on islands, with the lowest richness on small, remote islands, following species‐area and distance‐decay relationships (MacArthur and Wilson 1967). While the framework was developed for free‐living organisms, parasite distributions are assumed to behave similarly because parasites are closely associated with their hosts; therefore, island features will similarly constrain colonisation and extinction processes (Poulin and Morand 2000). Studies have shown the expected pattern of lower parasite diversity on islands for various ecto‐ and endoparasites in birds (Espinaze et al. 2019), amphibians (Belasen et al. 2019), fish (Dallarés et al. 2016) and mammals (Kornienko and Dokuchaev 2023). However, conflicting patterns have also been reported in a similarly broad range of parasites and hosts, including birds (Barrientos et al. 2014), amphibians (Aguiar et al. 2020) and insects (Votýpka et al. 2019). Even within one group of well‐studied parasites, avian haemosporidians, deviations from island biogeography expectations are frequently reported. For instance, variation in distance‐decay relationships has been observed among haemosporidian genera in South Pacific island white‐eyes (Zosteropidae) (Ishtiaq et al. 2010) and for haemosporidian lineage richness patterns in birds of the Gulf of Guinea (Loiseau et al. 2017). These deviations from island biogeography expectations are often attributed to differences in parasite life histories, such as life cycle complexity (Poulin et al. 2011; Williams et al. 2022), or level of host specialisation (Ishtiaq et al. 2010).

Transmission strategies are a potentially important life history trait to consider when aiming to understand patterns of parasite distributions. Parasites with simple life cycles, that is, those that require a single host, may transmit via host contact, faecal‐oral routes and environmental contamination (Antonovics et al. 2017). These transmission strategies impose varied constraints on parasite dispersal. Faecal‐oral or environmentally transmitted parasites can produce encysted stages, with a resistant outer layer that provides protection from adverse environmental conditions, such as temperature extremes and desiccation (Fayer 1980; Aguilar‐Díaz et al. 2011). Encysted stages can persist in the environment when suitable hosts are unavailable, increasing the chance of establishment (Guernier et al. 2004; Dunn et al. 2010). These dormant stages may also be dispersed via zoochory when attached to mobile animals (Coughlan et al. 2017). These parasites spend the majority of their replicating life cycle within a host (Fayer 1980) and are likely to co‐disperse with them, thus their patterns of dispersal may reflect that of their hosts (McGuinness 1984). As birds show strong adherence to island biogeography expectations (Valente et al. 2020), it follows that simple life cycle, environmentally transmitted parasites of birds will likewise show such patterns.

Parasites with complex life cycles which require multiple hosts are also characterised by a range of transmission strategies. Some are transmitted via bites from ticks or flying insect vectors; others are transmitted trophically, via consumption of additional infected hosts, or by skin penetration. Reliance on multiple hosts may make persistence more precarious as outcomes will depend on local availability of appropriate additional hosts or vectors (Lafferty 2012; Cizauskas et al. 2017; Wood et al. 2023). The ability of complex life cycle parasites to colonise islands may also be dependent on transmission strategy. For example, for parasites that utilise ticks as vectors, infected ticks may co‐disperse with hosts across extensive distances (Cohen et al. 2015; Sparagano et al. 2015; Loss et al. 2016). Colonisation of islands by parasites disseminated by flying insect vectors may depend on environmental factors such as offshore wind (Clark and Clegg 2015), while those transmitted trophically or via skin penetration, which require contact between hosts for transmission, will be dependent on the dispersal propensity of each host in the life cycle. Establishment of parasites with complex life cycles may also be more challenged by abiotic variation than is the case for environmentally transmitted parasites as both hosts must tolerate different conditions (Cable et al. 2017).

To date, most studies on the patterns of island biogeography in parasites have examined single parasite taxa (e.g., Maria et al. 2009; Santiago‐Alarcon et al. 2012; Olsson‐Pons et al. 2015; Padilla et al. 2017; Williamson et al. 2019; Amaya‐Mejia et al. 2022). This limits our understanding of how parasite life history traits, such as transmission strategy, may impact biogeographic patterns. However, characterising a wider diversity of parasites is challenging, particularly because specific host–parasite interactions are not well documented (Dallas et al. 2017; Farrell et al. 2022). This makes it difficult to select parasite taxa to target. In addition, identification of a broad range of taxa requires expertise in taxonomy, varied methodological approaches and can incur a high financial cost (Dobson et al. 2008; Okamura et al. 2018; De Buron et al. 2025). The ability of high‐throughput sequencing technologies to generate Whole Genome Sequence (WGS) data at scale (Hudson 2008) can ameliorate these challenges and offers a new avenue for characterising parasites. A relatively neglected feature of WGS data is that they can also capture information about inadvertently sequenced pathogens and parasites infecting the host (Kostic et al. 2011; Nichols et al. 2026). Therefore, in addition to addressing evolutionary questions about free‐living organisms, WGS data present an opportunity to simultaneously investigate endoparasites of the host. Utilising WGS data in this way enables the identification of a broad range of affiliated parasites with varied life histories (Nichols et al. 2026).

Here we use WGS data of individual silvereyes, *Zosterops lateralis*, a passerine species that has colonised many islands throughout the South Pacific (Mees 1969; Lack 1971; Estandía et al. 2025), to identify inadvertently sequenced parasites. Our aim is to characterise the distribution and diversity of such parasites in island and mainland silvereye populations to illustrate patterns of island biogeography with respect to richness and nestedness of parasite communities across host populations and to explore how life history strategies may modulate these patterns. We expect that distributions of parasites utilising environmental transmission will more closely reflect island biogeography patterns seen in free‐living organisms than those with complex life cycles utilising varied transmission strategies.

## 2 Materials and Methods

### 2.1 Study System

The silvereye is a small passerine bird distributed in Australia and islands of the south Pacific (Figure 1). Their distribution spans a latitudinal range of −14° to −47°. Island silvereye populations vary in age from hundreds to hundreds of thousands of years (Clegg et al. 2002; Estandía et al. 2025). They occur on islands ranging in size and geographic isolation, from the large Gondwanan islands of Grand Terre, New Caledonia and North and South Islands of New Zealand, to small volcanic islands, such as those of Vanuatu and coral cays, such as Heron Island on the Great Barrier Reef.

**FIGURE 1:**
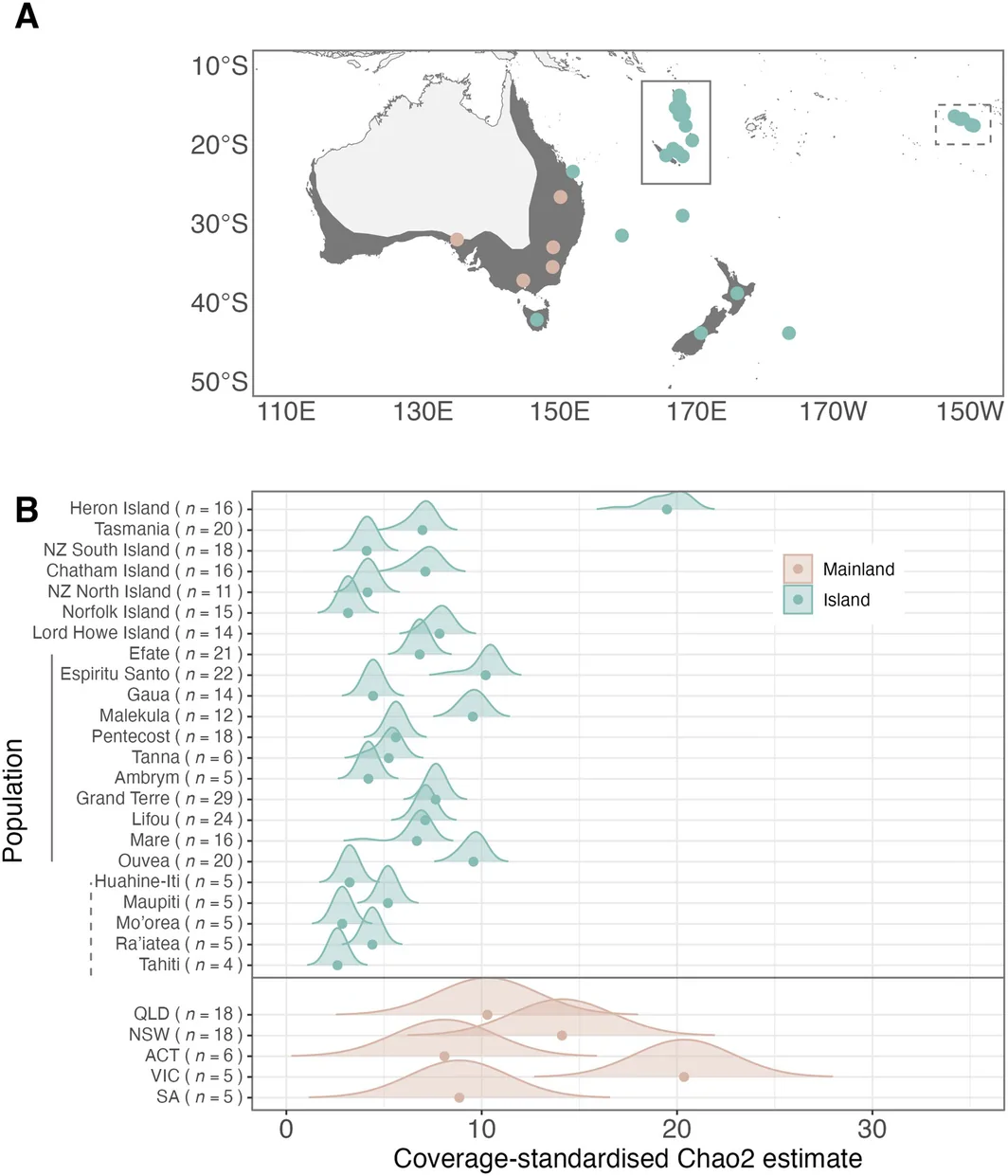
(A) Map of sampled continental mainland (beige) and island (turquoise) populations with dark grey shading indicating the sampled silvereye range. (B) coverage‐standardised Chao2 richness estimates (C = 0.80). Distributions show delete‐one‐jackknife estimates; populations with five or fewer individuals are shown as a single estimate.

### 2.2 Sample Collection, DNA Extraction and Sequencing

The blood samples used in this analysis are listed in Supplementary Appendix A. They were collected between 1996 and 2019 from 381 individual silvereyes spanning 25 South Pacific islands and five locations on the Australian continent (Queensland, New South Wales, Australian Capital Territory, South Australia and Victoria). Wild birds were caught using mist nets or traps. Blood was taken using a sterile needle to prick the brachial vein. Blood was collected in a glass capillary tube and placed in a 1.5 mL microfuge tube. The microfuge tubes contained Queen's Lysis Buffer modified from Seutin et al. (1991) with the final concentrations 0.01 M Tris, 0.01 M NaCl, 0.01 M EDTA and 0.03 M n‐lauroylsarcosine (10% w/v) and a pH of 7.5. Samples were kept at ambient temperature until they could be refrigerated and then frozen at −20°C.

DNA extractions were carried out using a modified phenol‐chloroform protocol (Sambrook and Russell 2006). Approximately 100 μL of blood preserved in Queen's Lysis Buffer was incubated overnight with 250 μL DIGSOL extraction buffer (0.02 M EDTA, 0.05 M Tris–HCl [pH 8.0], 0.4 M NaCl, 0.5% sodium dodecyl sulphate [SDS]) and 10 μL Proteinase K (20 mg/mL). Incubation occurred at 55°C with rotation. Following incubation, 250 μL phenol:chloroform:isoamyl alcohol (25:24:1) was added and samples were rotated for 10 min then centrifuged at 10,000 rpm for 10 min before transferring the aqueous phase to a new microfuge tube. This process was done twice more, first using another 250 μL phenol:chloroform:isoamyl alcohol (25:24:1), then using 250 μL chloroform:isoamyl alcohol (24:1). Two volumes of cold 100% ethanol, 1 volume of 2.5 M ammonium acetate and 1 μL glycogen were added to the final aqueous phase to precipitate the DNA. The samples were stored at −20°C for a minimum of 12 h before three rounds of centrifugation for 10 min at 15,000 rpm at 4°C. The supernatant was subsequently discarded and the precipitate washed with 500 μL cold 70% ethanol. The precipitated DNA was left to dry at room temperature. Once dried, the DNA was resuspended in 50 μL of TrisEDTA (TE) buffer (0.01 M Tris–HCl [pH 8.0], 0.0001 M EDTA). A negative extraction control was included for every batch of samples processed (maximum of 24 per batch).

DNA extractions were quantified with a Qubit 2.0 (Thermofisher Scientific Inc., United States) and were sent to Novogene UK or The Ramaciotti Centre for Genomics, Australia for library preparation and whole genome sequencing, aiming for a minimum of 5X coverage (Supplementary Appendix A; Figure S1) on the Illumina Novaseq 6000 platform (Illumina, San Diego) and generating 150 bp paired‐end reads. However, a number of samples were sequenced at a greater sequencing depth, up to 32X. While our previous work found that parasite genera detection was not sensitive to sequence depth beyond 5X (Nichols et al. 2026), it should also be noted that individuals sequenced at greater depth could have an inflated number of parasite genera detected.

### 2.3 Identification of Parasite Sequences

#### 2.3.1 Quality Control and Removal of Host Sequences

To characterise parasitic reads from the WGS data, the approach used by Nichols et al. (2026) was employed as follows. We used fastp (Chen et al. 2018), with default settings to remove adapter content (10 bp) and remove low‐quality reads (mean quality < *Q*15) from the raw sequence data. Host sequence removal was performed using the PathSeq pipeline from the Genome Analysis Toolkit (GATK) (Walker et al. 2018) using an existing *Z. lateralis* assembly (Estandía et al. 2023) as a reference. Sequences from each sample were compared to a k‐mer library generated from the *Z. lateralis* assembly using a fast k‐mer search. We used the default PathSeq settings to identify reads belonging to the host: reads were removed if there was a minimum of one k‐mer match to the host (Walker et al. 2018). The remaining reads were then aligned to a Burrows‐Wheeler Aligner (BWA)‐indexed host reference using the BWA‐MEM algorithm (Li 2013). Reads with an identity score above 30 were discarded.

Following host sequence removal, quality control was carried out with PathSeq. Reads with an excess of A/T or G/C content (29 out of 30 bases per window (Benjamini and Speed 2012)), duplicate sequences, and those shorter than 60 bp were discarded. Low‐complexity sequences and low‐quality bases were also masked using the sDUST algorithm (Morgulis et al. 2006). Read ends were further trimmed according to base quality (quality =*Q*15).

While we considered assembling longer contiguous sequences from the remaining sequences, our ability to detect certain parasites decreased following assembly (Supplementary Appendix B). This may be because some parasite DNA is likely to be present at extremely low levels in the host DNA and therefore will not have sufficient coverage for assembly and because parasite reference availability is limited for longer sequences. Therefore, we opted to use shorter sequences for classification but to impose a stringent confidence filter on the subsequent taxonomic assignments. The factors to consider when making decisions about extracting parasite sequences from WGS data of the host are outlined in full in Nichols et al. (2026).

#### 2.3.2 Building a Custom Reference Database of Eukaryotic Parasite Sequences

Reference database completeness is known to influence the outcome of taxonomic assignments (Hleap et al. 2021; Jeunen et al. 2023). Therefore, we compiled a custom reference database in Kraken 2 to maximise parasite coverage. We utilised the pre‐built bacteria, Protozoa, UniVec and human databases in Kraken 2. We then compiled additional reference sequences from various sources (Table S1). We downloaded all available genome references for apicomplexans, fornicata, cercozoans, parabasalids and euglenozoans from RefSeq (Pruitt et al. 2007; O'Leary et al. 2016). A complete list of NCBI taxonomic sequence identifiers was generated for the same taxonomic groups using TaxonKit (Shen and Ren 2021). We used Biopython to download the sequences from NCBI after removing any sequences labelled as ‘uncultured’ or ‘environmental’ (Cock et al. 2009; version released 05‐08‐2024). All genomic references from WormBase ParaSite (Howe et al. 2017) and the Eukaryotic Pathogen Genomics Database Resource (EuPathDB; Warrenfeltz et al. 2018) were also incorporated. Finally, the PR^2^ database was added following filtering to remove sequences labelled uncultured, environmental, fungal and metazoan. Kraken 2 derives a compact hash table of k‐mers from these reference sequences to be queried against during sequence assignment.

#### 2.3.3 Taxonomic Identification

Sequences were assigned using the custom parasite reference database with Kraken 2 (Wood et al. 2019). Following taxonomic assignment, we searched for information on previously recorded primary hosts and categorised the primary hosts for each parasite genus as bird, other vertebrate, invertebrate, or plant, accordingly. We removed parasites that have not previously been recorded in birds or other vertebrates (Supplementary Appendix C). For the detected parasite genera, we collated information from the literature on their life histories, which enabled assignment to the following transmission categories: parasites with complex life cycles that are transmitted by flying insect vectors (flying insect vector), parasites with complex life cycles that are carried by ticks (tick‐borne), parasites with complex life cycles that do not rely on flying insect vectors or ticks, which may be transmitted to the vertebrate host either trophically or via skin penetration (other complex life cycles), or environmentally transmitted parasites with simple life cycles that utilise faecal‐oral transmission (environmental). We also identified a single incidence of *Trichomonas* which is transmitted by host contact and two incidences of *Polystoma* which has a single host but is transmitted by skin penetration. In view of the low sample sizes of these parasites, both were excluded from the transmission strategy analyses. We were also unable to include *Olssonium* in our analysis as we were unable to find a record of its transmission strategy. In some cases, detected parasites employ ambiguous or variable transmission strategies. In such instances, we assigned life history on a case‐by‐case basis, considering the biology of our system. For example, we detected *Dipetalonema* which can be transmitted by either ticks or flying insect vectors. However, *Dipetalonema* has not yet been described in avian hosts; therefore, we assume that this detection represents a closely related avian filarioid nematode for which a reference sequence is not available. Avian filarioid nematodes are generally transmitted by insects (Bartlett 2008). Accordingly, we categorise *Dipetalonema* as being transmitted by flying insects. Supplementary Appendix D contains a summary of the identified parasites, including available life history information.

#### 2.3.4 Confidence Assignment

Confidence scores for each taxonomic assignment were generated using Conifer (Silamiķelism 2020). These were calculated according to the fraction of k‐mers assigned to the final taxonomic identity, its descendants and ascendants, giving the Root‐to‐Leaf (RTL) confidence score. We applied a confidence score filter of 0.7 for the paired‐end dataset. We previously found that applying a conservative confidence score to relatively short paired‐end sequences resulted in fewer apparently spurious parasite identifications, that is, fewer parasites that have not been previously reported in birds; and reasonably consistent identification of selected parasites (haemosporidians) while continuing to capture a large amount of diversity (Nichols et al. 2026).

### 2.4 Statistical Analysis

#### 2.4.1 Is Parasite Diversity Higher on the Mainland Compared to Islands?

Statistical analysis was conducted in R version 4.4.2 (R Core Team 2024). To test the island biogeography expectation of higher parasite richness in mainland compared to island populations, we estimated Chao2 richness per population. To account for both uneven sampling across populations as well as differences in sequence depth between the mainland and island samples, we used these Chao2 estimates for sample‐coverage‐based rarefaction and extrapolation (Chao and Jost 2012; Colwell et al. 2012; Chao et al. 2014), implemented in the iNEXT package version 3.0.2 (Hsieh et al. 2025). For each population, we calculated an incidence‐based rarefaction curve and standardised richness to a common target coverage of 0.80, fixed a priori. Standardising by coverage rather than by the number of hosts means that populations are compared at an equivalent level of sample completeness, even where sequencing depth differs. Ambae was excluded because it could not reach this target coverage, even when extrapolated to twice its observed sample size, Vanua Lava was also excluded from comparisons because the sample size was too small for reliable standardisation. To describe variation around each estimate, we used a delete‐one jackknife approach: for populations with more than five individuals, each individual was removed in turn and coverage‐standardised richness was recalculated from the remaining individuals, giving one estimate per individual removed. For populations with five or fewer individuals, a jackknife was not possible and the estimate from the full sample is reported. To test for an overall difference between mainland and island parasite richness, we performed a global site‐level permutation test, comparing the mean coverage‐standardised Chao2 estimate for mainland and island populations with 10,000 permutations. We randomly reassigned the mainland/island labels among populations while preserving the observed numbers of mainland and island sites to generate a null distribution of the difference in richness.

We tested whether the Australian continent (‘the mainland’) represents a regional source for parasite communities in the island populations by looking for evidence of community nestedness. The Nestedness metric based on Overlap and Decreasing Fill (NODF) was generated using the vegan package version 2.7.2 (Oksanen et al. 2025). To assess whether observed nestedness differed from that expected by chance alone, we compared our matrix to 999 simulations of a row‐constrained null model, in which parasite richness on each island (row totals) was conserved across the matrix to account for uneven sampling size, while parasite incidence (column totals) was randomised. We performed the analyses on two datasets: in the first, we included the mainland and each island population individually; and in the second, we combined data from islands within each of the French Polynesia and Southern Melanesia archipelagos. We then considered each archipelago as single units for analysis to reflect the geographical clustering of the islands.

#### 2.4.2 Comparing Mainland and Island Parasite Communities

To examine how variation in parasite communities differed in host individuals from the islands compared to the Australian continent, we implemented an extreme gradient boosting (XGBoost) classification algorithm using the XGBoost package 1.7.11.1 (Chen and Guestrin 2016). We aimed to test whether a host individual's origin (mainland or island) could be predicted according to a ‘parasite profile’. The parasite profiles consisted of the number of genera detected in each host individual per transmission category (flying insect vector, tick‐borne, other complex life cycle not involving insect vectors or ticks and environmental). The predicted variable was host origin (i.e., island or mainland). We subsequently extracted importance scores for each of the four transmission categories in classifying host individuals as mainland or island origin. Although XGBoost has been shown to work effectively on imbalanced datasets in the past (Zhao et al. 2018), it is nonetheless likely to cause a decline in model performance if one class is overrepresented (Wang et al. 2020). Therefore, we applied class weighting to the model to account for the imbalanced sample sizes in the island (*n* = 326) and mainland (*n* = 52) categories. Specifically, we used the ratio of mainland to island host individuals to adjust the gradient updates during training by increasing the weight of errors made on the underrepresented class (mainland hosts), thereby preventing the model from being biased toward the majority class (island hosts). We examined the distribution of probabilities output by the model to confirm that class imbalance was not causing bias in predictions (Figure S2). We used leave‐one‐out cross‐validation (LOOCV), where one host individual was withheld in each iteration of the model. We calculated Area Under the Receiver Operating Characteristic Curve (AUC ROC) scores to assess the model. To ensure that the differing read depth between samples or the total number of parasites per individual did not impact the performance of the model, we repeated the process using proportions of each category per bird.

To further illustrate the differences in parasite communities on islands and the continental mainland, we compared parasite richness and prevalence separately for each transmission category. For each transmission category, we estimated Chao2 per population using the bias‐corrected estimator implemented in the vegan package. We drew 1000 random subsamples of five individuals without replacement from each population (all individuals were used for populations with *n* ≤ 5), calculated Chao2 for each subsample and took the mean across subsamples as the estimate for that population. We did not apply sample‐coverage standardisation to these analyses because among transmission categories, several populations had too few detections for sample coverage to be estimated reliably. We compared mainland and island populations using a site‐level permutation test with 10,000 permutations. During each permutation, mainland/island labels were randomly reassigned among populations while preserving the overall numbers of mainland and island sites to generate a null distribution of the difference in mean Chao2 richness. An equivalent site‐level permutation test was used to compare parasite prevalence between mainland and island populations for each transmission category. In both cases, we adjusted for multiple comparisons using a Bonferroni correction.

#### 2.4.3 Is Parasite Richness Higher on Less Isolated, Larger Islands?

To test whether parasite richness increased with island size and decreased with island isolation, we fitted a generalised linear model (GLM) and a generalised linear mixed model (GLMM) to compare Chao2 richness among islands. For each model, we used the same set of predictor variables: island area, island isolation and latitude. To calculate island area and isolation, we utilised the Natural Earth 10 m landmass data (Kelso and Patterson 2010; Pebesma 2018). Areas were computed on the WGS84 ellipsoid (EPSG:4326). We considered two metrics of isolation to capture larger‐scale and regional‐level isolation. First, we calculated the distance to mainland as the shortest possible distance between the coast of mainland Australia and each island (km). Second, we followed the procedure outlined by Itescu et al. (2020) and Weigelt and Kreft (2013), generating a concentric 100 km buffer around each island and calculating the area of landmass it contained, excluding the island itself. This captures a more regional isolation level, which is relevant when a dataset comprises both single isolated islands and islands that fall within archipelagos. Isolation metrics and area varied across several orders of magnitude. To reduce positive skew, we applied a log_10_ transformation to island area and both measures of isolation prior to modelling. To control for the possibility that parasite communities vary according to latitude (Nunn et al. 2005; Poulin and Leung 2011), latitude was included as a covariate in the models. Our sampling took place over two decades, over which time parasite communities may be liable to change (Wood et al. 2023). However, we were unable to include sampling year as a random effect because most islands were sampled in a single year; therefore, including year as a random factor would prevent models from converging. The models were weighted by the number of individuals sampled in each population so that richness estimates derived from larger samples contributed proportionally more to model fitting than estimates based on smaller sample sizes.

To avoid inclusion of collinear predictor variables in the models, pairwise Pearson correlation coefficients were calculated between each predictor variable to ensure that none exceeded a correlation threshold of *r* > 0.7, which they did not. Generalised variance inflation factors (GVIFs) were also computed using the *car* package to quantify multicollinearity (Fox and Weisberg 2019). The moderate correlation between proximate island isolation and island area led to elevated GVIF scores. Therefore, we excluded regional isolation and recalculated VIF scores (Table S2). Our GLMM includes interaction terms between categorical and continuous variables; therefore, we mean‐centred the continuous variables prior to calculating GVIFs (Aiken and West 1991; Robinson and Schumacker 2009). We report estimates based on mean‐centred values in our models. All standardised scores were below two, indicating acceptable levels of collinearity for inclusion in the model.

In the GLM, we used log_10_‐transformed mean estimates for Chao2 richness per island as the response variable and specified a Gaussian distribution of errors. In the GLMM, we classified parasite genera into environmental, flying insect vector transmitted parasites or complex life cycle parasites that do not rely on flying insects or ticks for transmission and calculated Chao2 richness for each category. We excluded tick‐borne parasites from this analysis as the overall richness detected was low (only two genera were detected) and Chao2 estimates were thus unlikely to be informative (Colwell and Coddington 1994). Chao2 richness for each island (total and per transmission category) was estimated by repeated subsampling of five individuals without replacement (1000 subsamples; see above). We did not use sample‐coverage standardisation here because these models include island populations only and so are not affected by the difference in sequencing depth between island and mainland samples. We found that using a log_10_‐transformation was not sufficient to adjust the right‐skewed data, so we instead used a gamma distribution of errors to accommodate the resultant non‐negative, count‐like richness measures. Despite being count‐like, Chao2 is in fact a bias‐corrected estimate derived from incidence data (Gotelli and Colwell 2001). Therefore, Chao2 estimates are continuous, non‐integer values which cannot be modelled using error distributions that are commonly used for count data. Gamma distributions require positive values; therefore islands in which no parasite genera of a given transmission strategy were detected were excluded for that category. This applied to four islands (Ambae, Moorea, Raiatea and Tahiti). We included island as a random factor to account for non‐independence of Chao2 estimates from the same population. The inclusion of this random factor may also help to account for temporal variation in the data as many islands were sampled during a single year. Following model fitting, we used the DHARMa package (Hartig and Hartig 2017) to assess model fit (Figure S3).

For both models, we performed model selection and averaging using the MuMIn package (Barton and Barton 2015). All candidate models were compared based on Akaike's Information Criterion corrected for small sample sizes (AICc). Models within ΔAICc < 3 of the top‐ranked model were considered equally plausible and we generated model‐averaged predictions across this top model set. Model selection tables are included in Tables S3 and S4.

To ensure that our results were not driven by any individual island, we conducted a leave‐one‐out sensitivity analysis. This was conducted by refitting the GLMM with each island excluded in turn and comparing the resulting isolation × transmission strategy coefficients to those from the full model (Table S5).

## 3 Results

We characterised 46 parasite genera from whole genome sequencing data belonging to 378 of 381 individual silvereye (no parasite genera passed filtering in three individuals). Of the parasites that could be attributed a transmission category, the majority had complex life cycles that do not require a flying insect vector or tick (44%), followed by those transmitted by flying insect vectors (28%), environmentally transmitted genera (23%) and lastly by tick‐borne parasite genera (5%). Of the four groups, flying insect vector‐transmitted parasites had the highest prevalence.

### 3.1 Is Parasite Richness Higher on the Mainland Compared to Islands?

Parasite richness was generally higher on the mainland than for island populations, with the global site‐level permutation test indicating higher richness across mainland populations (mean coverage‐standardised Chao2: mainland = 12.34, island = 6.44; difference = 5.90, *p =* 0.007). Although most island populations had lower Chao2 estimates than the mainland, Heron Island was notable for having a substantially higher, albeit highly variable, estimated Chao2 richness compared to the mainland mean (Figure 1).

The parasite communities of host populations exhibited significant nestedness (Figure 2). In the analysis considering each island population separately, the NODF was 66.40, substantially higher than expected under the null model (null model mean = 48.90, 95% CI = 44.8–52.8, *p* < 0.001). This corresponds to a standardised effect size of 8.17. The mainland fell at the apex of the nestedness structure, with island parasite communities forming subsets of the genera found on the mainland (Figure 2A). However, it should be noted that differences in sequence depth between the mainland and island populations could have impacted these results. In the analysis that considered islands within Southern Melanesia and French Polynesia at the archipelago level, the observed NODF was 73.11 (null model: mean = 60.0, 95% CI = 53.09–66.30, *p* < 0.001) and the standardised effect size was 4.03. However, in this analysis, Southern Melanesia fell at the apex of the nestedness structure (Figure 2B).

**FIGURE 2:**
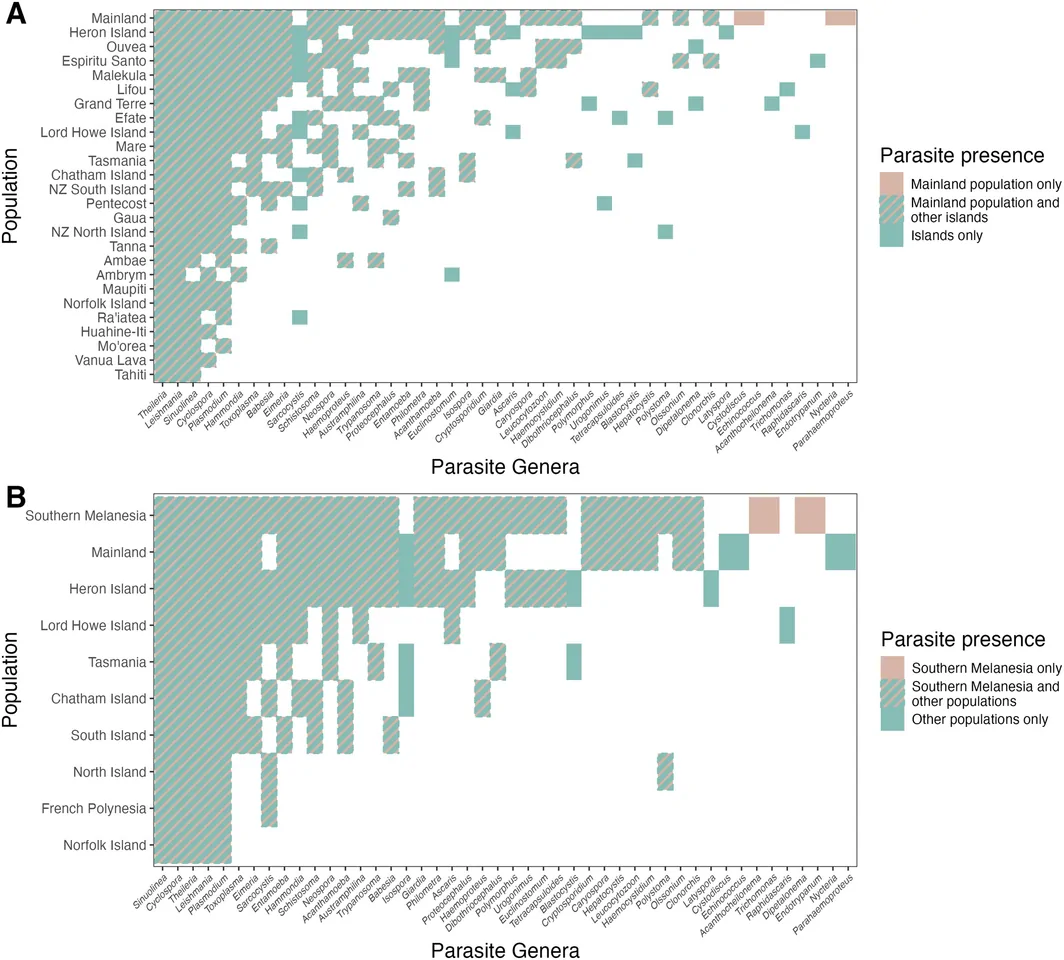
Nestedness structure among (A) the mainland and each island population and (B) the mainland, single islands and the Southern Melanesia and French Polynesia archipelagos (grouping islands within each of these archipelagos).

### 3.2 Comparing Mainland and Island Parasite Communities

According to LOOCV, the XGBoost model had a classification accuracy of 81% and an AUC ROC score of 86%, indicating that the parasite profile, that is, the number of parasite genera detected per individual host per transmission category, provides discriminatory power to identify host samples sourced from an island versus the mainland. The AUC ROC score remained at 85% when proportions of each parasite category per individual was used instead, indicating that total number of parasites per individual did not significantly impact predictive power of the model. Feature importance scores extracted from the model indicated that flying insect vector‐transmitted parasites were most informative in this distinction (Figure 3A). Furthermore, the mean Chao2 richness was significantly higher for mainland than island populations for environmentally transmitted (mean Chao2 difference = 2.257, *p = 0.024*) and flying insect vector‐transmitted parasites (mean Chao2 difference = 3.119, *p = 0.008*) whereas there was no significant difference for parasites transmitted by ticks (mean Chao2 difference = 0.242, *p =* 0.601) or complex life cycle parasites not requiring flying insects or ticks for transmission (mean Chao2 difference = 3.149, *p =* 0.282; Figure 3B). The prevalence of complex life cycle parasites that do not require flying insects or ticks for transmission was higher on the mainland than in the island populations (difference in prevalence: 0.318, *p =* 0.024). No difference was found between island and mainland populations for flying insect vector (difference in prevalence: 0.077, *p =* 0.961), tick‐borne (difference in prevalence: 0.243, *p =* 0.074) or environmentally transmitted parasites (difference in prevalence: 0.220, *p =* 0.351).

**FIGURE 3:**
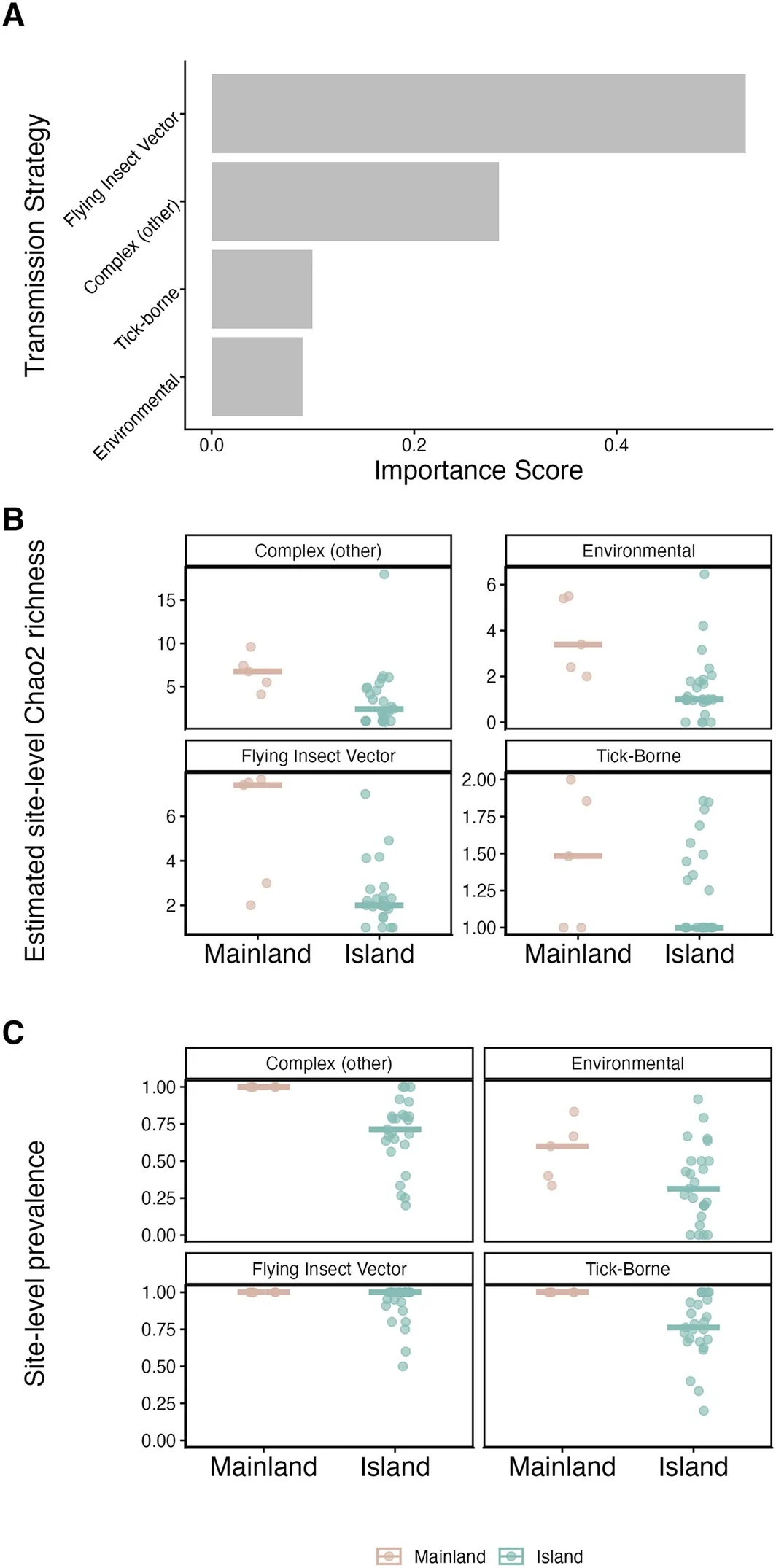
(A) Importance scores extracted from XGBoost when classifying 378 individual hosts into island and mainland origins according to their parasite profile (number of parasite genera per transmission category detected per individual host) (B) Estimated Chao2 detected per population according to transmission category, (C) prevalence of parasites detected per population according to transmission category. Crossbars represent mean for island and mainland populations.

### 3.3 Is Parasite Richness Higher on Less Isolated, Larger Islands?

For total Chao2 richness per island, two models were averaged based on AICc (Table 1). Island area was included in one of the two models (Table S3) and because the models were similarly weighted, island area was attributed a weighted importance score of 0.45, while latitude and isolation from the Australian continent were attributed weighted importance scores of 1. While there was support for including all three variables in the models, isolation from the Australian continent had the most substantial effect size, with richness decreasing as isolation from the Australian continent increased (Figure 4B). Although island area and latitude did explain some of the variation in the data, it was not possible to confidently discern the direction of the relationships.

**TABLE 1:** Model‐averaged estimates for GLM predicting total Chao2 richness based on parasite genera detected per island population.

Parameter	1	2	*β*	SE	95% CI	WIP
Intercept	•	•	2.437	0.085	(2.271, 2.603)	
Latitude	•	•	0.014	0.009	(−0.004, 0.031)	1.00
log10 continental island isolation (km)	•	•	−0.378	0.107	(−0.588, −0.169)	1.00
log10 Island area (km^2^)		•	−0.013	0.030	(−0.048, 0.036)	0.45
df	4.00	5.00				
logLik	−942.63	−941.24				
AICc	1895.26	1895.65				
Delta	0.00	0.38				
Weight	0.55	0.45				

*Note:* Estimates, standard errors, 95% CI and weighted importance scores (WIP) are based on model averaging across the top two models (ΔAICc < 3; Table S3). The parameters included in each model (1 and 2) are indicated by black dots; for each model in the top set, we also present degrees of freedom (df), Log‐likelihood (logLik), the difference in AICc from the top model (delta) and the relative support for each model according to the AICc scores (weight).

**FIGURE 4:**
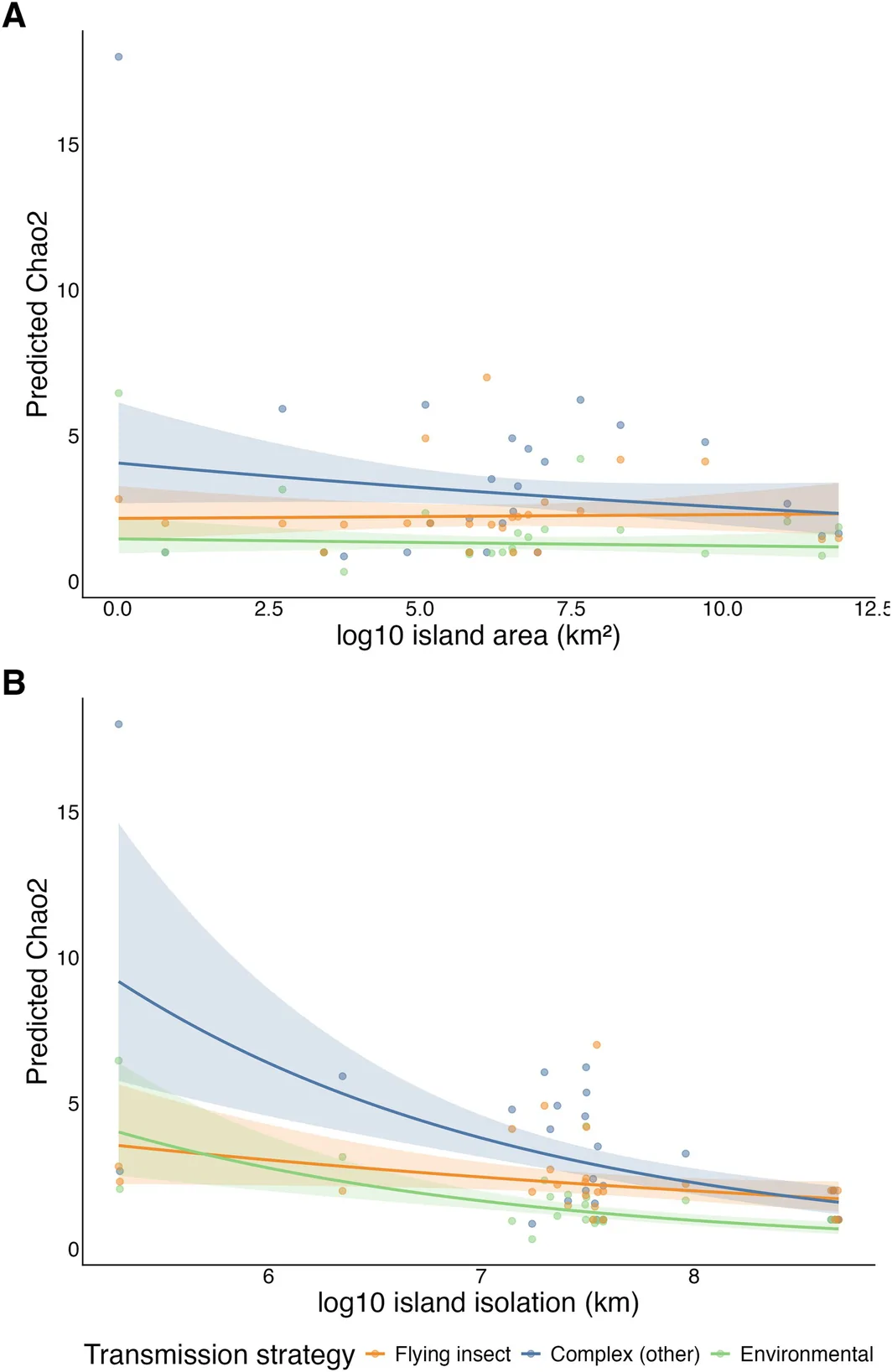
Predicted Chao2 richness as a function of (A) area and (B) isolation from the Australian continent for Chao2 richness per island per transmission strategy. Predictions are based on an average of the top two models with < 3 AICc. Points represent observed island population values used to generate predictions.

For Chao2 richness per transmission category on each island, two models were averaged based on AICc scores (Table S4). Parasite richness declined with isolation from the Australian continent overall and we found that the relationship between Chao2 richness and isolation from the Australian continent was dependent on transmission strategy (Figure 4B). Distance had a much weaker impact on parasites that rely on flying insect vectors. However, there were substantial declines in richness for parasites using environmental transmission strategies and those with complex life cycles not using flying insects or ticks for transmission. It was not possible to discern the direction of the relationship between Chao2 richness and island area, but the interaction between island area and transmission strategy indicated that parasites with complex life cycles that did not require a flying insect or tick for transmission declined with island area while those utilising environmental transmission were slightly negative and there was no detectable effect for parasites utilising flying insect vectors for transmission. We found substantial variation between Chao2 richness for each transmission category: at the mean island area and continental isolation observed in our dataset, parasites with complex life cycles that do not involve a flying insect or tick had the highest predicted Chao2 richness, followed by those transmitted by flying insect vectors and finally environmentally transmitted parasites, which had the lowest predicted richness. Latitude was included in one of the two top models and was attributed a weighted importance score of 0.32. The directionality of this effect could not be confidently inferred as the 95% CI encompassed zero (Table 2).

**TABLE 2:** Model‐averaged estimates for GLMMs predicting Chao2 richness per transmission category on each of 25 islands.

Parameter	1	2	β	SE	95% CI	WIP
Intercept	•	•	0.807	0.084	(0.642, 0.973)	
log10 Island area (km^2^)	•	•	0.006	0.031	(−0.056, 0.067)	1.00
log10 continental island isolation (km)	•	•	−0.214	0.104	(−0.418, −0.009)	1.00
Complex (other)	•	•	0.302	0.026	(0.251, 0.352)	1.00
Environmental	•	•	−0.540	0.027	(−0.593, −0.487)	1.00
log10 Island area (km^2^) × Complex (other)	•	•	−0.052	0.009	(−0.069, −0.035)	1.00
log10 Island area (km^2^) × Environmental	•	•	−0.023	0.008	(−0.040, −0.007)	1.00
log10 continental island isolation (km) × Complex (other)	•	•	−0.303	0.032	(−0.365, −0.241)	1.00
log10 continental island isolation (km) × Environmental	•	•	−0.310	0.031	(−0.371, −0.249)	1.00
Latitude		•	0.012	0.010	(−0.012, 0.020)	0.32
Random effect: Island	Variance = 0.155; SD = 0.394; levels = 25					
df	11.00	12.00				
logLik	−1086.57	−1085.90				
AICc	2199.62	2201.18				
Delta	0.00	1.55				
Weight	0.68	0.32				

*Note:* Estimates, standard errors, 95% CI and weighted importance scores (WIP) are based on the top model set (ΔAICc < 3; Table S4). The parameters included in each model are indicated by black dots; for each model in the top set, we also present degrees of freedom (df), Log‐likelihood (logLik), the difference in AICc from the top model (delta) and the relative support for each model according to the AICc scores (weight). Area = log_10_ mean‐centred Island Area (km^2^), Isolation = log_10_ mean‐centred isolation from the Australian continent (km), TS = Transmission Strategy. Island was included as a random effect.

## 4 Discussion

By extracting parasite DNA from low‐coverage whole genome sequences of hosts, we empirically demonstrate how parasite distributions reflect the expected patterns of island biogeography. Notably, we found that distribution patterns were differentially driven by parasites utilising varied life history strategies. Overall, parasite richness was higher on mainland Australia compared to island populations although the strength of this effect varied with parasite transmission strategies and among island populations, parasite richness decreased with distance from the continental mainland although this pattern also varied according to life history strategy. Taken together with our nestedness analysis, it appears that parasite diversity in the region is sourced from both the Australian continent and Melanesian archipelagos and highlights that variation in parasite life history strategies shapes these patterns of diversity.

### 4.1 Isolation and Parasite Life History Traits Influence Parasite Richness and Prevalence

Our results clearly demonstrate that transmission strategies influence patterns of island biogeography for parasites. For environmentally transmitted parasites with simple life cycles, we found lower parasite richness in island populations, with richness declining with distance from the continental mainland. These results could indicate that when the primary dispersal mechanism is co‐dispersal with the host, island isolation acts as a dispersal filter simultaneously for both host and parasite. Such patterns have been described in macroparasites with direct life cycles (Williams et al. 2022; Nieberding et al. 2006). However, this explanation most likely applies to highly host‐specific parasites, which cannot disperse via alternative routes. Unfortunately, we are unable to quantify host specificity in the present study. An alternative explanation may be that hosts on the mainland experience greater exposure to human and livestock waste, elevating opportunities for infection by faecal‐orally transmitted parasites. Previous work found that parasites infecting humans decreased in richness with distance from the mainland (Jean et al. 2016). However, evidence that human populations show some of the expected patterns of island biogeography is lacking (Mologni and Burns 2023). Therefore, it is unclear what the mechanism underpinning this pattern may be.

Parasites with complex life cycles showed varied patterns of island biogeography. Those that utilise complex life cycles but are not transmitted by dispersive intermediate hosts are expected to be more limited in their establishment success (Cizauskas et al. 2017; Wood et al. 2023) and this should lead to stronger patterns of island biogeography. Indeed, we found an isolation by distance effect for parasites with complex life cycles that are not transmitted by flying insect vectors or ticks. However, we detected no significant difference overall in richness between island versus mainland populations for these parasites. A possible explanation for this pattern is that utilisation of a paratenic host (a host that can harbour the parasite in a dormant state) may facilitate persistence in newly colonised environments as parasites do not require immediate availability of definitive hosts (Morand et al. 1995; Parker et al. 2015; Cable et al. 2017). Therefore, rather than being a limitation, intermediate host use may enhance opportunities for colonisation and persistence on islands, although dispersal opportunities for such intermediate hosts would still be expected to diminish with increasing island isolation.

Parasites transmitted by flying insect vectors exhibited a reduction in genus richness on islands compared to the mainland, yet this initial reduction in richness did not decline further with increasing island isolation. The overall reduction in richness suggests that islands impose a strong barrier to colonisation for parasites transmitted by flying insect vectors. This follows expectations as successful transmission requires a suitable vertebrate host and compatible flying insect vector (Spurgin et al. 2012). Variation in the dispersal propensities and flight mechanisms of flying insects (Compton 2002), combined with the relatively limited availability of habitat types on islands to support flying insect vector populations (McGuinness 1984; Turner and Tjørve 2005), may restrict diversity of vectors able to establish on islands, thereby constraining the diversity of vector‐dependent parasites (Robert et al. 2011). Accordingly, strong isolation‐by‐distance patterns may not be expected if dispersal is limited or absent even at low levels of isolation. It has been previously established that some vectors are capable of traversing long distances. For example, wind‐assisted dispersal of *Anopheles mosquitos* can promote travel over 300 km in a 9 h flight period (Huestis et al. 2019). For the subset of parasites whose vectors can traverse long distances to colonise islands, increasing island isolation likely exerts a weak influence on colonisation success. Understanding the impact of island isolation on parasites transmitted by flying insect vectors has important implications for future disease projections under climate change.

In contrast to parasite richness, prevalence of flying insect vector‐transmitted parasites was similar and relatively high in both the mainland and island populations. While a reduction in prevalence may have been expected on islands, such high prevalence of island parasites has been reported previously (Lindenfors et al. 2007; Illera et al. 2015; Dybing et al. 2018) and reflects a well‐established biogeographic trend for free‐living organisms whereby the subset of species that occur on islands often reach higher population densities (MacArthur et al. 1972). Maintenance of comparable parasite prevalence on islands (cf mainland) may therefore be supported by high host densities, which facilitate frequent transmission opportunities (Fromont et al. 2001; De Bellocq et al. 2002).

### 4.2 Island Area and Parasite Richness

Although area was important in explaining differences in parasite richness among islands, we did not detect a positive correlation between richness and island area, as expected under the rules of island biogeography. As described above, island populations often experience density compensation, whereby population density is higher than might be expected on a similar‐sized area on the mainland due to reduced competition (MacArthur et al. 1972; Wright 1980). For parasites, habitat availability is effectively represented by host availability. Therefore, if small islands have a high host population density, the amount of habitat available to parasites may remain comparable to that available on larger islands. High host density on the smallest islands may increase host individual contact rates, driving up transmission opportunities (Fromont et al. 2001; De Bellocq et al. 2002). Frequent contact between definitive and intermediate hosts is likely to have a stronger impact on parasites that are transmitted by predator–prey interactions or skin penetration than those that are transmitted by independently dispersive vectors such as flying insects. Increased host density may also explain the small increase in richness on smaller islands for parasites with complex life cycles that do not rely on flying insects or ticks for transmission.

Furthermore, while our study included some very large islands, for example, the North and South Islands of New Zealand, which are ~114,000 and ~150,000 km^2^, respectively, most of the islands included would be considered small according to the UNESCO definition (< 2000 km^2^; Falkland and Custodio 1991). The lack of a positive correlation between richness and island area may reflect the Small Island Effect (SIE), which describes how the relationship between species richness and island area can be decoupled for small islands (Lomolino and Weiser 2001). The mechanisms underlying this effect include reduced habitat diversity on small islands (Sfenthourakis and Triantis 2009; Chen et al. 2020), edge effects (Lomolino and Weiser 2001), or disequilibrium between colonisation and extinction events. Heron Island (0.29 km^2^) exemplifies the decoupling described by the SIE. Despite being the smallest in our dataset, the population hosted the richest parasite community of the islands in the dataset. The proximity of Heron Island to the mainland (~80 km) likely bolsters the parasite community via the relatively frequent arrival of a wide variety of vagrant birds (Kikkawa 1970) and vectors (Clark and Clegg 2015). The position of Heron Island immediately below mainland Australia in the nestedness analysis supports the scenario that parasites readily colonise from the mainland. The high parasite richness of Heron Island silvereyes may be further elevated by sharing of parasites from the high‐density breeding populations of Black Noddies ( *Anous minutus* ) and Wedge‐tailed Shearwaters (*Ardenna pacifica*) (Hill et al. 1997; Dyer et al. 2005). Several parasite genera detected on Heron Island are known to parasitise seabirds, for example, *Sarcocystis* (Gjerde et al. 2018; Sato et al. 2022), *Euclinostomum* (Abro et al. 2016; Shamsi et al. 2021) and *Polymorphus* (Kuklina and Kuklin 2021; Galaktionov and Atrashkevich 2023). Therefore, for small islands, stochastic processes and ecological factors may have a greater impact on richness than area‐dependent relationships predicted by classical island biogeography.

### 4.3 Sources of Parasite Fauna

The nestedness analysis identified the Australian mainland as the dominant source of parasites for the sampled island populations. However, the detection of several parasite genera on the islands but not on the Australian mainland indicates that multiple source populations may be involved in determining regional parasite assemblages. When analysed at the archipelago scale, Southern Melanesia occurred at the apex of the hierarchical structure of nestedness. New Guinea is a major source for much of the regional avifauna in Southern Melanesia (Diamond 1970; Filardi and Moyle 2005; Jønsson et al. 2010; Jønsson et al. 2011) and it follows that movements of birds from New Guinea have provided a source of parasite diversity in Southern Melanesia and beyond.

Host colonisation history likely further influences the parasite assemblages detected across islands. Silvereyes have colonised islands throughout the South Pacific over a range of timescales. Some populations in Southern Melanesia are estimated to have colonised hundreds of thousands of years ago, while others are the product of recent, human‐mediated introductions. For instance, silvereyes in French Polynesia exhibit low parasite richness. These populations were established from an introduction of a small number of silvereyes from South Island New Zealand in the 1930s (Guild 1940; Sendell‐Price et al. 2020) and the low parasite richness observed may reflect failure to colonise with the host, or early loss post‐colonisation (MacLeod et al. 2010). In addition, parasite gain from other avifauna is likely minimal. The native avifauna may have had historically low parasite diversity due to the highly isolated nature of the archipelago. However, non‐native introductions that occurred in the late 19th and early 20th centuries (Thibault and Cibois 2017) conceivably provide opportunities for parasite sharing and, with time, new host–parasite relationships in French Polynesian silvereyes may arise.

### 4.4 Caveats

Many other facets of parasite life history may also influence patterns of biogeography. For example, host breadth and specificity may impact the likelihood of colonisation success. De Bellocq et al. (2002) found that host specificity of parasites significantly decreased on islands when comparing the outcomes of 28 studies on 16 species of mammal. Therefore, it is likely that being a host generalist confers a benefit to parasites colonising islands. Indeed, each transmission category in the present study is likely to encompass a range of host use patterns such as *Toxoplasma*, which are known to be flexible in terms of host use (Boothroyd 2009). However, there is limited understanding of the breadth of host associations and patterns of host use for many parasites, making it challenging to empirically assess how host specificity influences parasite distributions across islands. There has been a sustained interest in characterising the degree of host specificity for some avian parasites (Bensch et al. 2000; Schrenzel et al. 2005; Križanauskienė et al. 2006; Schoener et al. 2013; Clark and Clegg 2017). Such work may help to elucidate the impact of host specialisation on island colonisation in the future.

Re‐purposing WGS data to characterise affiliated parasites involves some trade‐offs. While the approach enables detection of a broad range of parasites at the genus level, some diversity may be overlooked due to the limited availability of published reference sequences. Furthermore, the lack of longer or complete genome reference sequences means that assembly of short reads into longer contiguous sequences yields fewer matches. Therefore, we used short sequences despite the possibilities of assignment to an incorrect reference or the inclusion of sequencing errors that could inflate the richness estimates we report here. To combat this issue, we applied a stringent confidence filter for identifications and classified only to the genus level (Nichols et al. 2026). Our approach thus reflects the decision to maximise diversity capture, despite the likely incorporation of some false‐positive identifications.

Parasite communities are likely to change over time. These changes may take place over decades (Wood et al. 2023) or seasonally (Ferreira Junior et al. 2017). Our sampling spanned two decades and most island populations were sampled within a single field season. Given this data structure, we were unable to account for temporal variation in our analyses. This is a common challenge in field studies conducted across broad spatial scales because logistical constraints often necessitate sampling different locations in different years. Changes in parasite communities over short and long‐term scales could confound the detected effects in our study. Future longitudinal sampling of the same populations would be required to fully disentangle temporal from spatial patterns in parasite community composition.

## 5 Conclusion

By leveraging existing WGS data from an island‐colonising bird, we provide evidence that large‐scale parasite distributions follow the rules of island biogeography, but these distributions are mediated by aspects of parasite life history. This suggests that inconsistent biogeographical patterns revealed in previous studies that focus on particular parasite groups (e.g., Illera et al. 2015; Jean et al. 2016; Votýpka et al. 2019) can be at least partially explained by variation in life history, an insight that is only revealed by examining a broad range of parasites. While the increasing availability of parasite sequence data in public databases will enhance analyses of distribution patterns, a concerted effort is also needed to gather information on parasite ecology and life history traits (Cizauskas et al. 2017). Such integrative data will enable a more nuanced understanding of parasite distributions over space and improve forecasts of future distributional changes, including the spread of disease agents.

## Author Contributions

Conceptualisation: Sarah Nichols, Sonya M. Clegg and Beth Okamura designed the research. Investigation: Sonya M. Clegg conducted fieldwork in Southern Melanesia, French Polynesia, New Zealand, Australia (including Tasmania, Norfolk Island, Lord Howe Island and Heron Island); Bruce C. Robertson and Fiona Robertson conducted fieldwork in New Zealand and Australia (including Tasmania); Andrea Estandía and Fiona Robertson conducted the lab work. Formal analysis: Sarah Nichols analysed the data and visualised the data. Supervision: Sonya M. Clegg and Beth Okamura provided supervisory oversight. Writing, original draft: Sarah Nichols wrote the paper with Beth Okamura and Sonya M. Clegg. Writing, review and editing: All authors provided feedback on the manuscript during the drafting process.

## Funding

We thank the funders of this work: the Department of Biology, Oxford and Oxford University John Fell Fund grant (ATD00330) to SMC to support sequencing; Marsden Fund New Zealand (14‐UOO‐231) to BCR and SMC to support fieldwork on mainland Australia and Heron Island and sequencing; National Geographic Society Committee for Research and Exploration Grant (9383‐13) to SMC to support fieldwork in New Caledonia; Natural Environment Research Council (NERC) fellowship (NE/C518381/1) to SMC to support fieldwork in Vanuatu and New Caledonia; Percy Sladen Memorial Fund (Linnean Society of London) to SMC to support fieldwork in French Polynesia; SN was supported by a NERC studentship (NE/S007474/1); AE was supported by a NERC studentship (NE/S007474/1) and a St John's College Graduate Scholarship.

## Ethics Statement

Ethics clearances were provided by University of Queensland ethics committee (ZOO/165/94/ARC, ZOO/520/96/ARC/PHD, ZOO/520/97/ARC/PHD), Griffith University ethics committee (ENV/01/12/AEC, ENV/07/16/AEC, ENV/06/20/AEC, ENV/24/13/AEC) to SMC, Charles Sturt University AEC to BCR and M. Massaro, and Otago University AEC to BCR.

## Conflicts of Interest

The authors declare no conflicts of interest.

## Supporting information


**Supplementary Appendix A:** Metadata for *Zosterops lateralis* samples collected and used to investigate the impact of parasite life histories on patterns of biogeography. Each row represents a single sample (*n* = 381) and each column provides information about host taxonomy, collection location, collection date, and sequencing platform.
**Supplementary Appendix B:** Exhaustive list of genera detected following assembly of short fragments into longer contiguous sequences.
**Supplementary Appendix C:** List of genera that were removed from the final dataset and the reason for removal.
**Supplementary Appendix D:** Summary of the identified parasites retained in the analyses, including available life history information.

**Table S1:** Summary of reference sequences included in the custom Kraken 2 database.
**Table S2:** Pairwise Variance inflation factors (VIFs) and Pearson correlation coefficients calculated for each predictor variable included in the GLMs.
**Table S3:** Model selection results for GLM predicting total Chao2 richness based on parasite genera detected per island population. Each row represents an individual model and includes parameter estimates (intercept and coefficients), degrees of freedom (df), log‐likelihood (logLik), Akaike Information Criterion corrected for small sample size (AICc), delta AICc (ΔAICc) and Akaike weights. NA values indicate that the parameter was not included in the model. Area = log_10_ Island Area (km^2^), Isolation = log_10_ isolation from the Australian continent (km).
**Table S4:** Model selection results for GLMM predicting Chao2 richness based on parasite genera categorised under each transmission category detected per island population. Each row represents an individual model and includes parameter estimates (intercept and coefficients), degrees of freedom (df), log‐likelihood (logLik), Akaike Information Criterion corrected for small sample size (AICc), delta AICc (ΔAICc) and Akaike weights. NA values indicate that the parameter was not included in the model. Area = log_10_ Island Area (km^2^), Isolation = log_10_ isolation from the Australian continent (km), TS = Transmission Strategy. Island was included as a random factor.
**Table S5:** Leave‐one‐out sensitivity analysis for the isolation from the Australian continent × transmission strategy interaction term in the GLMM predicting Chao2 richness per transmission category. Each row shows the re‐estimated coefficient (β) and 95% confidence interval for the two comparisons made: Complex versus flying insect vector and Environmental versus flying insect vector after refitting the model and excluding an island.
**Figure S1:** Distribution of mean genome coverage (depth) for 381 individual silvereye whole genome sequences.
**Figure S2:** Distribution of predicted probabilities output from XGBoost when classifying 378 individual silvereye into island and mainland origin groups according to their parasite profile (number of parasite genera per transmission category detected per individual host).
**Figure S3:** Residual plots for (A) GLM predicting total Chao2 richness based on parasite genera detected per island population and (B) GLMM predicting total Chao2 richness based on parasite genera detected per island population per transmission mode.

## Data Availability

Raw sequence reads are available in the Sequence Read Archive (SRA; BioProject: PRJNA1554506). The associated sample metadata will also be stored in the SRA. The sample information is available as Supplementary Material. The code used to analyse the data is available on GitHub: https://github.com/sarah‐nichols/island‐biogeography‐wgs‐mining.git. Benefits from this research accrue from the sharing of our data and approach on public databases as described above.
