# Supplementary material for: Transmission Strategy Modulates Parasite Biogeography in an Island‐Colonising Bird
Source: Mol Ecol. 2026 Nov 13;35(22):e70659. doi: 10.1111/mec.70659 (PMC13768733; doi:10.1111/mec.70659)
Supplement: Supplementary file 1 [file MEC-35-e70659-s002.docx]

**Table S1.** Summary of reference sequences included in the custom Kraken 2 database.

| Source | Details | Sequences obtained (n) |
| --- | --- | --- |
| NCBI Genomes | Downloaded via the NCBI Datasets tool, including RefSeq for: | |
|  | [Apicomplexans](https://www.ncbi.nlm.nih.gov/datasets/genome/?taxon=5794) | 372 |
|  | [Cercozoans](https://www.ncbi.nlm.nih.gov/datasets/genome/?taxon=136419) | 5 |
|  | [Euglenozoa](https://www.ncbi.nlm.nih.gov/datasets/genome/?taxon=33682) | 188 |
|  | [Fornicata](https://www.ncbi.nlm.nih.gov/datasets/genome/?taxon=207245) | 36 |
|  | [Parabasalids](https://www.ncbi.nlm.nih.gov/datasets/genome/?taxon=5719) | 10 |
| [WormBase ParaSite](https://parasite.wormbase.org/ftp.html) | Version: WBPS18 | 4,856,896 |
| [EuPathDB](https://veupathdb.org/veupathdb/app/fasta-tool/genomic-sequence) | Release 64 12 Jul 2023 | 281,024 |
| [PR^2^ database](https://pr2-database.org/) | Release 5.0.0 with metazoan, fungal and uncultured sequences removed. | 101,252 |
| [NCBI Nucleotide database](https://www.ncbi.nlm.nih.gov/nuccore) | All available sequences for apicomplexans, cercozoans, euglenozoa, fornicata and parabasalids (See supplementary material for TaxIDs). | 37,153 |

**Figure S1.** Distribution of mean genome coverage (depth) for 381 individual silvereye whole genome sequences.

**Figure S2.** Distribution of predicted probabilities output from XGBoost when classifying 378 individual silvereye into island and mainland origin groups according to their parasite profile (number of parasite genera per transmission category detected per individual host).

**Table S2.** Pairwise Variance inflation factors (VIFs) and Pearson correlation coefficients calculated for each predictor variable included in the GLMs.

| **Variable** | **Df** | **GVIF** | **GVIF^(1/2Df)** | **Island area** | **Regional isolation** | **Continental isolation** | **Latitude** | **Population size** |
| --- | --- | --- | --- | --- | --- | --- | --- | --- |
| **Island area** | 1 | 3.22 | 1.79 | 1.00 | 0.66 | -0.09 | -0.43 | 0.35 |
| **Regional isolation** |  |  |  |  | 1.00 | 0.24 | 0.13 | -0.03 |
| **Continental isolation** | 1 | 3.04 | 1.74 |  |  | 1.00 | 0.40 | -0.51 |
| **Latitude** | 1 | 1.39 | 1.18 |  |  |  | 1.00 | -0.32 |
| **Population size** | 1 | 1.55 | 1.67 |  |  |  |  | 1.00 |
| **Transmission Strategy** | 2 | 1.26 | 1.06 |  |  |  |  |  |
| **Island area × Transmission strategy** | 2 | 3.15 | 1.33 |  |  |  |  |  |
| **Isolation × Transmission strategy** | 2 | 3.23 | 1.34 |  |  |  |  |  |

**A**

**B**

**Figure S3.** Residual plots for A) GLM predicting total Chao2 richness based on parasite genera detected per island population and B) GLMM predicting total Chao2 richness based on parasite genera detected per island population per transmission mode.

**Table S3.** Model selection results for GLM predicting total Chao2 richness based on parasite genera detected per island population. Each row represents an individual model and includes parameter estimates (intercept and coefficients), degrees of freedom (df), log-likelihood (logLik), Akaike Information Criterion corrected for small sample size (AICc), delta AICc (ΔAICc), and Akaike weights. NA values indicate that the parameter was not included in the model. Area = log_10_ Island Area (km²), Isolation = log_10_ isolation from the Australian continent (km).

| Intercept | Area | Latitude | Isolation | | Df | Log Likelihood | | AICc | ΔAICc | Weight | |
| --- | --- | --- | --- | --- | --- | --- | --- | --- | --- | --- | --- |
| 2.43 | NA | 0.01 | | -0.39 | 4 | -942.63 | 1895.26 | | 0.00 | 0.55 |  |
| 2.44 | -0.01 | 0.01 | | -0.37 | 5 | -941.24 | 1895.65 | | 0.38 | 0.45 |  |
| 2.45 | -0.03 | NA | | -0.32 | 4 | -955.95 | 1921.90 | | 26.64 | 0.00 |  |
| 2.43 | NA | NA | | -0.35 | 3 | -962.69 | 1932.52 | | 37.26 | 0.00 |  |
| 2.57 | -0.05 | NA | | NA | 3 | -1009.28 | 2025.69 | | 130.43 | 0.00 |  |
| 2.57 | -0.05 | 0.00 | | NA | 4 | -1009.11 | 2028.23 | | 132.96 | 0.00 |  |
| 2.56 | NA | 0.01 | | NA | 3 | -1028.45 | 2064.04 | | 168.78 | 0.00 |  |
| 2.55 | NA | NA | | NA | 2 | -1031.47 | 2067.48 | | 172.22 | 0.00 |  |
| 2.43 | NA | 0.01 | | -0.39 | 4 | -942.63 | 1895.26 | | 0.00 | 0.55 |  |
| 2.44 | -0.01 | 0.01 | | -0.37 | 5 | -941.24 | 1895.65 | | 0.38 | 0.45 |  |
| 2.45 | -0.03 | NA | | -0.32 | 4 | -955.95 | 1921.90 | | 26.64 | 0.00 |  |
| 2.43 | NA | NA | | -0.35 | 3 | -962.69 | 1932.52 | | 37.26 | 0.00 |  |
| 2.57 | -0.05 | NA | | NA | 3 | -1009.28 | 2025.69 | | 130.43 | 0.00 |  |
| 2.57 | -0.05 | 0.00 | | NA | 4 | -1009.11 | 2028.23 | | 132.96 | 0.00 |  |
| 2.56 | NA | 0.01 | | NA | 3 | -1028.45 | 2064.04 | | 168.78 | 0.00 |  |
| 2.55 | NA | NA | | NA | 2 | -1031.47 | 2067.48 | | 172.22 | 0.00 |  |

**Table S4.** Model selection results for GLMM predicting Chao2 richness based on parasite genera categorised under each transmission category detected per island population. Each row represents an individual model and includes parameter estimates (intercept and coefficients), degrees of freedom (df), log-likelihood (logLik), Akaike Information Criterion corrected for small sample size (AICc), delta AICc (ΔAICc), and Akaike weights. NA values indicate that the parameter was not included in the model. Area = log_10_ Island Area (km²), Isolation = log_10_ isolation from the Australian continent (km), TS = Transmission Strategy. Island was included as a random factor.

| Intercept | Area | Latitude | Isolation | TS | Area x TS | Isolation x TS | Df | Log Likelihood | AICc | ΔAICc | Weight |
| --- | --- | --- | --- | --- | --- | --- | --- | --- | --- | --- | --- |
| 0.81 | 0.00 | NA | -0.20 | + | + | + | 11 | -1086.57 | 2199.62 | 0.00 | 0.68 |
| 0.81 | 0.02 | 0.01 | -0.25 | + | + | + | 12 | -1085.90 | 2201.18 | 1.55 | 0.32 |
| 0.82 | NA | NA | -0.21 | + | NA | + | 8 | -1105.44 | 2229.19 | 29.57 | 0.00 |
| 0.82 | NA | 0.01 | -0.26 | + | NA | + | 9 | -1104.44 | 2229.84 | 30.22 | 0.00 |
| 0.82 | -0.02 | NA | -0.21 | + | NA | + | 9 | -1105.10 | 2231.15 | 31.52 | 0.00 |
| 0.82 | -0.01 | 0.01 | -0.26 | + | NA | + | 10 | -1104.41 | 2232.50 | 32.87 | 0.00 |
| 0.79 | -0.01 | NA | -0.40 | + | + | NA | 9 | -1146.30 | 2313.56 | 113.93 | 0.00 |
| 0.79 | 0.01 | 0.01 | -0.44 | + | + | NA | 10 | -1145.63 | 2314.92 | 115.30 | 0.00 |
| 0.77 | 0.00 | NA | NA | + | + | NA | 8 | -1152.73 | 2323.78 | 124.15 | 0.00 |
| 0.77 | -0.01 | -0.01 | NA | + | + | NA | 9 | -1152.61 | 2326.17 | 126.55 | 0.00 |
| 0.81 | NA | NA | -0.40 | + | NA | NA | 6 | -1159.70 | 2332.70 | 133.08 | 0.00 |
| 0.81 | NA | 0.01 | -0.46 | + | NA | NA | 7 | -1158.59 | 2332.96 | 133.34 | 0.00 |
| 0.81 | -0.03 | NA | -0.41 | + | NA | NA | 7 | -1159.16 | 2334.09 | 134.47 | 0.00 |
| 0.81 | -0.01 | 0.01 | -0.46 | + | NA | NA | 8 | -1158.49 | 2335.30 | 135.67 | 0.00 |
| 0.79 | NA | NA | NA | + | NA | NA | 5 | -1165.96 | 2342.84 | 143.21 | 0.00 |
| 0.79 | -0.02 | NA | NA | + | NA | NA | 6 | -1165.81 | 2344.94 | 145.32 | 0.00 |
| 0.79 | NA | 0.00 | NA | + | NA | NA | 6 | -1165.93 | 2345.18 | 145.55 | 0.00 |
| 0.79 | -0.03 | -0.01 | NA | + | NA | NA | 7 | -1165.68 | 2347.15 | 147.52 | 0.00 |
| 0.81 | NA | 0.02 | -0.46 | NA | NA | NA | 5 | -1488.90 | 2988.73 | 789.11 | 0.00 |
| 0.81 | NA | NA | -0.38 | NA | NA | NA | 4 | -1490.41 | 2989.42 | 789.80 | 0.00 |
| 0.81 | -0.03 | NA | -0.39 | NA | NA | NA | 5 | -1489.82 | 2990.56 | 790.94 | 0.00 |
| 0.81 | -0.01 | 0.02 | -0.45 | NA | NA | NA | 6 | -1488.83 | 2990.97 | 791.34 | 0.00 |
| 0.79 | NA | NA | NA | NA | NA | NA | 3 | -1495.77 | 2997.90 | 798.27 | 0.00 |
| 0.79 | -0.02 | NA | NA | NA | NA | NA | 4 | -1495.58 | 2999.76 | 800.14 | 0.00 |
| 0.79 | NA | 0.00 | NA | NA | NA | NA | 4 | -1495.77 | 3000.14 | 800.52 | 0.00 |
| 0.79 | -0.03 | 0.00 | NA | NA | NA | NA | 5 | -1495.55 | 3002.02 | 802.40 | 0.00 |
| 0.81 | 0.00 | NA | -0.20 | + | + | + | 11 | -1086.57 | 2199.62 | 0.00 | 0.68 |
| 0.81 | 0.02 | 0.01 | -0.25 | + | + | + | 12 | -1085.90 | 2201.18 | 1.55 | 0.32 |
| 0.82 | NA | NA | -0.21 | + | NA | + | 8 | -1105.44 | 2229.19 | 29.57 | 0.00 |
| 0.82 | NA | 0.01 | -0.26 | + | NA | + | 9 | -1104.44 | 2229.84 | 30.22 | 0.00 |
| 0.82 | -0.02 | NA | -0.21 | + | NA | + | 9 | -1105.10 | 2231.15 | 31.52 | 0.00 |
| 0.82 | -0.01 | 0.01 | -0.26 | + | NA | + | 10 | -1104.41 | 2232.50 | 32.87 | 0.00 |
| 0.79 | -0.01 | NA | -0.40 | + | + | NA | 9 | -1146.30 | 2313.56 | 113.93 | 0.00 |
| 0.79 | 0.01 | 0.01 | -0.44 | + | + | NA | 10 | -1145.63 | 2314.92 | 115.30 | 0.00 |
| 0.77 | 0.00 | NA | NA | + | + | NA | 8 | -1152.73 | 2323.78 | 124.15 | 0.00 |
| 0.77 | -0.01 | -0.01 | NA | + | + | NA | 9 | -1152.61 | 2326.17 | 126.55 | 0.00 |
| 0.81 | NA | NA | -0.40 | + | NA | NA | 6 | -1159.70 | 2332.70 | 133.08 | 0.00 |
| 0.81 | NA | 0.01 | -0.46 | + | NA | NA | 7 | -1158.59 | 2332.96 | 133.34 | 0.00 |
| 0.81 | -0.03 | NA | -0.41 | + | NA | NA | 7 | -1159.16 | 2334.09 | 134.47 | 0.00 |
| 0.81 | -0.01 | 0.01 | -0.46 | + | NA | NA | 8 | -1158.49 | 2335.30 | 135.67 | 0.00 |
| 0.79 | NA | NA | NA | + | NA | NA | 5 | -1165.96 | 2342.84 | 143.21 | 0.00 |
| 0.79 | -0.02 | NA | NA | + | NA | NA | 6 | -1165.81 | 2344.94 | 145.32 | 0.00 |
| 0.79 | NA | 0.00 | NA | + | NA | NA | 6 | -1165.93 | 2345.18 | 145.55 | 0.00 |
| 0.79 | -0.03 | -0.01 | NA | + | NA | NA | 7 | -1165.68 | 2347.15 | 147.52 | 0.00 |
| 0.81 | NA | 0.02 | -0.46 | NA | NA | NA | 5 | -1488.90 | 2988.73 | 789.11 | 0.00 |
| 0.81 | NA | NA | -0.38 | NA | NA | NA | 4 | -1490.41 | 2989.42 | 789.80 | 0.00 |
| 0.81 | -0.03 | NA | -0.39 | NA | NA | NA | 5 | -1489.82 | 2990.56 | 790.94 | 0.00 |
| 0.81 | -0.01 | 0.02 | -0.45 | NA | NA | NA | 6 | -1488.83 | 2990.97 | 791.34 | 0.00 |
| 0.79 | NA | NA | NA | NA | NA | NA | 3 | -1495.77 | 2997.90 | 798.27 | 0.00 |
| 0.79 | -0.02 | NA | NA | NA | NA | NA | 4 | -1495.58 | 2999.76 | 800.14 | 0.00 |
| 0.79 | NA | 0.00 | NA | NA | NA | NA | 4 | -1495.77 | 3000.14 | 800.52 | 0.00 |
| 0.79 | -0.03 | 0.00 | NA | NA | NA | NA | 5 | -1495.55 | 3002.02 | 802.40 | 0.00 |
| 0.81 | 0.00 | NA | -0.20 | + | + | + | 11 | -1086.57 | 2199.62 | 0.00 | 0.68 |
| 0.81 | 0.02 | 0.01 | -0.25 | + | + | + | 12 | -1085.90 | 2201.18 | 1.55 | 0.32 |
| 0.82 | NA | NA | -0.21 | + | NA | + | 8 | -1105.44 | 2229.19 | 29.57 | 0.00 |
| 0.82 | NA | 0.01 | -0.26 | + | NA | + | 9 | -1104.44 | 2229.84 | 30.22 | 0.00 |
| 0.82 | -0.02 | NA | -0.21 | + | NA | + | 9 | -1105.10 | 2231.15 | 31.52 | 0.00 |
| 0.82 | -0.01 | 0.01 | -0.26 | + | NA | + | 10 | -1104.41 | 2232.50 | 32.87 | 0.00 |
| 0.79 | -0.01 | NA | -0.40 | + | + | NA | 9 | -1146.30 | 2313.56 | 113.93 | 0.00 |
| 0.79 | 0.01 | 0.01 | -0.44 | + | + | NA | 10 | -1145.63 | 2314.92 | 115.30 | 0.00 |
| 0.77 | 0.00 | NA | NA | + | + | NA | 8 | -1152.73 | 2323.78 | 124.15 | 0.00 |
| 0.77 | -0.01 | -0.01 | NA | + | + | NA | 9 | -1152.61 | 2326.17 | 126.55 | 0.00 |
| 0.81 | NA | NA | -0.40 | + | NA | NA | 6 | -1159.70 | 2332.70 | 133.08 | 0.00 |
| 0.81 | NA | 0.01 | -0.46 | + | NA | NA | 7 | -1158.59 | 2332.96 | 133.34 | 0.00 |
| 0.81 | -0.03 | NA | -0.41 | + | NA | NA | 7 | -1159.16 | 2334.09 | 134.47 | 0.00 |
| 0.81 | -0.01 | 0.01 | -0.46 | + | NA | NA | 8 | -1158.49 | 2335.30 | 135.67 | 0.00 |
| 0.79 | NA | NA | NA | + | NA | NA | 5 | -1165.96 | 2342.84 | 143.21 | 0.00 |
| 0.79 | -0.02 | NA | NA | + | NA | NA | 6 | -1165.81 | 2344.94 | 145.32 | 0.00 |
| 0.79 | NA | 0.00 | NA | + | NA | NA | 6 | -1165.93 | 2345.18 | 145.55 | 0.00 |
| 0.79 | -0.03 | -0.01 | NA | + | NA | NA | 7 | -1165.68 | 2347.15 | 147.52 | 0.00 |

**Table S5.** Leave-one-out sensitivity analysis for the isolation from the Australian continent × transmission strategy interaction term in the GLMM predicting Chao2 richness per transmission category. Each row shows the re-estimated coefficient (β) and 95% confidence interval for the two comparisons made: Complex vs. flying insect vector and Environmental vs. flying insect vector after refitting the model and excluding an island.

| **Excluded island** | **Complex β** | **Complex 95% CI** | **Environmental β** | **Environmental 95% CI** |
| --- | --- | --- | --- | --- |
| None (full model) | -0.303 | (-0.365, -0.241) | -0.310 | (-0.371, -0.249) |
| Ambae | -0.295 | (-0.353, -0.236) | -0.306 | (-0.364, -0.248) |
| Ambrym | -0.307 | (-0.368, -0.246) | -0.313 | (-0.373, -0.254) |
| Chatham | -0.318 | (-0.381, -0.255) | -0.339 | (-0.402, -0.277) |
| Efate | -0.316 | (-0.378, -0.254) | -0.313 | (-0.374, -0.251) |
| Gaua | -0.298 | (-0.360, -0.236) | -0.305 | (-0.366, -0.243) |
| Grand Terre | -0.292 | (-0.349, -0.235) | -0.315 | (-0.372, -0.258) |
| Heron Island | -0.104 | (-0.181, -0.028) | -0.125 | (-0.200, -0.049) |
| Huahine | -0.296 | (-0.357, -0.235) | -0.352 | (-0.413, -0.290) |
| Lifou | -0.304 | (-0.367, -0.241) | -0.311 | (-0.373, -0.249) |
| Lord Howe Island | -0.279 | (-0.343, -0.215) | -0.266 | (-0.328, -0.203) |
| Malekula | -0.312 | (-0.372, -0.253) | -0.331 | (-0.389, -0.273) |
| Mare | -0.309 | (-0.370, -0.248) | -0.305 | (-0.365, -0.245) |
| Maupiti | -0.269 | (-0.330, -0.208) | -0.320 | (-0.382, -0.258) |
| Moorea | -0.275 | (-0.337, -0.213) | -0.298 | (-0.358, -0.237) |
| Norfolk Island | -0.304 | (-0.357, -0.251) | -0.310 | (-0.362, -0.258) |
| North Island | -0.306 | (-0.368, -0.244) | -0.314 | (-0.375, -0.254) |
| Ouvea | -0.303 | (-0.365, -0.240) | -0.307 | (-0.368, -0.246) |
| Pentecost | -0.315 | (-0.377, -0.253) | -0.303 | (-0.365, -0.242) |
| Raiatea | -0.303 | (-0.366, -0.240) | -0.309 | (-0.370, -0.249) |
| Santo | -0.304 | (-0.366, -0.241) | -0.300 | (-0.362, -0.238) |
| South Island | -0.322 | (-0.381, -0.264) | -0.336 | (-0.393, -0.280) |
| Tahiti | -0.306 | (-0.369, -0.244) | -0.312 | (-0.372, -0.252) |
| Tanna | -0.301 | (-0.363, -0.240) | -0.309 | (-0.370, -0.249) |
| Tasmania | -0.543 | (-0.623, -0.463) | -0.370 | (-0.454, -0.285) |
| Vanua Lava | -0.302 | (-0.363, -0.241) | -0.312 | (-0.372, -0.252) |
